# Do Mimic Hoverflies Spatially Match Their Bumblebee Models? Evidence From a Polymorphic Mimicry System

**DOI:** 10.1002/ece3.74146

**Published:** 2026-08-11

**Authors:** Blessing Chidinma Umeh, Markus A. K. Sydenham, Michael A. Patten

**Affiliations:** ^1^ Ecology Research Group, Faculty of Bioscience and Aquaculture Nord University Steinkjer Norway; ^2^ Norsk Institutt for Naturforskning (NINA) Oslo Norway

**Keywords:** Batesian mimicry, bumblebees, co‐occurrence, hoverfly, polymorphism, spatial mismatch, species distribution

## Abstract

Batesian mimicry enables harmless species to evade predation by resembling defended models. Although mimicry theory predicts that mimics should broadly co‐occur with their models, increasing evidence suggests that mimic–model associations may extend beyond individual model species, and it remains unclear whether polymorphic mimics spatially track individual model species or broader communities of defended prey across landscapes. We used species distribution models based on presence and pseudoabsence data to quantify the geographic distributions and spatial overlap of three hoverfly mimics ( *Volucella bombylans* ) morphs and three putative bumblebee (*Bombus* sp.) model groups across Norway and tested whether mimic occurrences were more strongly associated with their putative models than with alternative *Bombus* species. All three hoverfly morphs showed spatial overlap with *Bombus* model groups. Occurrence of *V. b. “plumata”* and *V. b. “bombylans”* was positively associated with habitat suitability of their putative *Bombus* models and other *Bombus* species. However, *Bombus lapidarius* sensu lato showed the strongest spatial association with both morphs, whereas *V. b. “haemorrhoidalis”* showed no clear association with any *Bombus* species. Overall, our results do not support strict pairwise spatial associations between *V. bombylans* morphs and their *Bombus* models. Instead, they suggest that polymorphic *V. bombylans* morphs associate with broad *Bombus* assemblages rather than individual model species. This pattern is consistent with Batesian mimicry operating at the level of broader mimicry communities, where predator generalization across shared warning signals may reduce selection for strict model‐specific spatial associations.

## Graphical abstract

Polymorphic Batesian mimics are often expected to track the distributions of their respective model species. We found substantial overlap among color morphs of *Volucella bombylans* and their putative Bombus models, while habitat suitability for *Bombus lapidarius* positively predicted the occurrence of all three hoverfly morphs. Our findings suggest that morph distributions respond to the broader Bombus assemblage rather than to strict one‐to‐one associations with individual model species.

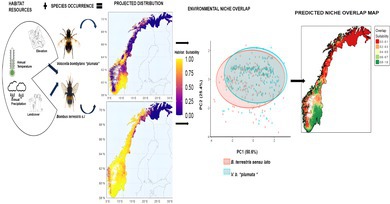

## 1 Introduction

Species coexistence is predicated on how organisms share space and time. Batesian mimicry illustrates one such interaction: harmless species evolve warning signals that resemble defended (toxic or noxious) models (Bates 1862), exploiting predator learning to reduce their own risk of attack. Survival of mimics increases in the presence of models (Brower 1960; Pfennig et al. 2001; Finkbeiner et al. 2018), although the degree to which mimics are protected depends critically on predator cognition. Because predators learn and forget warning signals over time (Speed 2000; Sherratt 2008), mimics experience increased predation when avoidance decays or when encounters with defended models are too infrequent to reinforce it.

Predator learning depends strongly on time, relative abundance, and spatial co‐occurrence of models and mimics (Mostler 1934; Mappes et al. 2005; Sherratt 2008). Theory therefore predicts close temporal and spatial overlap to ensure that predators encounter defended models frequently enough to maintain aversion to both models and mimics (Ruxton et al. 2019). Although Batesian mimics are rarely found entirely outside the ranges of their models, many systems contain populations occurring in both sympatry and partial allopatry with defended models (Pfennig et al. 2001; Prudic and Oliver 2008; Ries and Mullen 2008; Pfennig and Mullen 2010; Harper and Pfennig 2007), suggesting that mimic–model associations may vary geographically across landscapes, challeging the expectation of strict sympatry.

Mimics may resemble different model species across different parts of their geographic range (Mallet et al. 1999; Prudic and Oliver 2008; Kristiansen et al. 2018; see also Ruxton et al. 2019). Geographic variation in mimic–model associations may arise (Pfennig et al. 2001; Pfennig and Mullen 2010; Akcali and Pfennig 2017) because predator responses vary with local model‐mimic frequency (Pfennig, et al. 2007) (Iserbyt et al. 2011), predator‐mediated selection varies among prey communities (Kokko et al. 2003; Lindström et al. 2004; Bond 2007), or predators retain learned avoidance after local model loss (Akcali and Pfennig 2014). We therefore ask whether broad‐scale distributions of Batesian mimics are structured primarily by their putative model species or by broader communities of defended prey. Quantifying spatial associations between mimics and candidate model species therefore test whether mimic distributions conform to the expectation of close model–mimic sympatry.

The hoverfly *Volucella bombylans* (Diptera: Syrphidae) is one of the best‐studied examples of a polymorphic Batesian mimicry system. In hoverfly–bumblebee mimicry systems, visually oriented avian predators are thought to impose frequency‐dependent selection that favors resemblance to defended *Bombus* species (Mostler 1934; Dittrich et al. 1993; Penney et al. 2012). *Volucella bombylans* exhibits several color morphs that resemble common European bumblebees (*Bombus* spp.) in body coloration and pubescence (Stubbs and Falk 1983; Edmunds and Reader 2014). In northern Europe, the black‐and‐red *V. b. bombylans* morph resemble *Bombus lapidarius* , the yellow‐banded *V. b. plumata* morph resemble members of the *Bombus terrestris* complex, and the brownish *V. b. haemorrhoidalis* morph mimics *Bombus pascuorum* (Edmunds and Reader 2014). However, these associations are unlikely to be exclusive. For example, red‐tailed bumblebee species such as *B. ruderarius* and *B. sylvarum* in lowland habitats, and *B. lapponicus* , *B. monticola* , and *B. wurflenii* in boreal or alpine regions, may also represent plausible candidate models based on their similar warning colouration and regional occurrence for the red‐abdominal tip *V. bombylans* morph. Common European *Bombus* species frequently co‐occur across broad geographic regions despite differences in resource use at finer spatial scales (Edmunds and Reader 2014; Goulson et al. 2008; Goulson and Darvill 2004). This overlap makes it difficult to infer mimic–model associations from patterns of co‐occurrence alone and raises the question of whether hoverfly morphs are associated primarily with their traditional putative models or with broader *Bombus* assemblages (Edmunds and Reader 2014). Although each *V. bombylans* morph has traditionally been linked to a particular *Bombus* species (Edmunds and Reader 2014), it is unclear whether these associations hold across the species' geographic range.

Despite extensive work describing polymorphism and putative mimic–model relationships in bumblebee‐mimicking hoverflies (Edmunds 2000; Howarth and Edmunds 2000; Howarth et al. 2000, 2016; Edmunds and Reader 2014), few studies have examined whether these associations persist across broad geographic scales. Previous studies primarily tested mimicry predictions using local morph frequencies, predator responses, or abundance patterns (Edmunds and Reader 2014; Howarth et al. 2016). For example, Edmunds and Reader (2014) demonstrated that local morph frequencies reflected the relative abundance of some putative *Bombus* models at sites where predators were likely to encounter both mimics and model. Whether these local associations are maintained across the broader geographic distributions of hoverfly morphs and their candidate *Bombus* models remains unknown. Because occurrence records often provide incomplete and spatially biased representations of species distributions, species distribution models (SDMs) provide a robust framework for estimating geographic distributions, quantifying spatial overlap, and evaluating spatial associations between mimics and their candidate models (Peterson et al. 2011; Phillips et al. 2009).

Here, we used species distribution models to estimate the distributions of *V. bombylans* morphs and their putative *Bombus* models across Norway. We then examined whether hoverfly morphs were spatially associated with their presumed models at broad geographic scales. Specifically, we asked: (1) how do the distributions of hoverfly morphs and their putative *Bombus* models overlap across regions of sympatry, partial allopatry, and allopatry; and (2) are hoverfly morphs more strongly associated with their putative *Bombus* models than with alternative *Bombus* species? By addressing these questions, we evaluated whether broad‐scale spatial associations in this polymorphic Batesian mimicry system conform to the traditional expectation of one‐to‐one mimic–model relationships.

## 2 Methods

### 2.1 Data Extraction and Modeling

We compiled occurrence data for bumblebees and hoverflies from the Norwegian citizen science platform Artsdatabanken (https://www.artsdatabanken.no). The dataset was filtered to include only species identified as bumblebee models for the polymorphic hoverfly mimic *V. bombylans* (Edmunds 2008; Edmunds and Reader 2014; Howarth and Edmunds 2000; Speight et al. 2020; Van Veen 2014). We excluded entries that lacked valid species names, duplicate records, spatial coordinates (latitude and longitude), or had unclear photographs. *Volucella bombylans* exhibits three color morphs, so we reviewed images associated with each record to note, where possible, the observed color morph. Additionally, specimens from the Natural History Museums at the University of Oslo, University of Bergen, and Norwegian University of Science and Technology in Trondheim were examined to verify color morph variants. Because most *Bombus* models are cryptic species, and their mimics resemble the color patterns rather than the taxonomic identities of the species, we grouped *Bombus* species into color space groups (Edmunds 2008; Edmunds and Reader 2014; Howarth and Edmunds 2000; Van Veen 2014). We defined *Bombus terrestris sensu lato* to include *B. lucorum* , *B. cryptarum* , *B. magnus* , and *B. terrestris* ; *Bombus pascuorum sensu lato* to include *B. humilis* , *B. muscorum* , *B. consobrinus* and *B. hypnorum* , *B. cingulatus* and *B. pascuorum* ; and *Bombus lapidarius sensu lato* to include *B. ruderarius* , *B. sylvarum* , *B. wurflenii* , and *B. lapidarius* . This approach ensures that analyses capture ecologically relevant variation in color patterns rather than fine‐scale taxonomic distinctions among apparent cryptic species.

Each occurrence record included the observation date, species identity (including color morph classification), and geographic coordinates (latitude and longitude). We retained records from 1970 onward to match temporal coverage of WorldClim v2.1 bioclimatic variables (1970–2000; Fick and Hijmans 2017). We used annual mean temperature (Bio1) and annual precipitation (Bio12) as predictors of species distributions. We complemented these with elevation data from the WorldClim digital elevation model and land‐cover data from the NIBIO AR50 dataset (Norwegian Institute of Bioeconomy Research), which provided detailed habitat information. The *ar50* vector layer was rasterized at 1‐km resolution and reclassified into eight land‐cover categories: Built‐up, Agriculture, Forest, Snowfield, Bog, Glacier, Freshwater, and Ocean. To reduce spatial and environmental sampling bias, we applied the thin() function from the spThin package (Aiello‐Lammens et al. 2015), retaining a single occurrence per 1 km grid cell. This step reduced clustering in oversampled areas and minimized potential spatial bias in model calibration. Although this does not fully address spatial autocorrelation in the model, it does help to reduce potential spatial biases.

We implemented a custom function to define species‐specific geographic extents (on the basis of environmental layers), occurrence records, and dispersal constraints in the species‐specific study area. The function generated a raster mask representing the potential study region by combining several spatial filters: (i) a mask polygon to limit analyses to Norway, (ii) an elevation range of 0–1200 m reflecting known species limits in Norway, and (iii) species‐specific buffers around occurrence points representing estimated dispersal capacity. Buffer radii were set to 3 km for *V. bombylans* (including named color morphs *plumata, bombylans* and *haemorrhoidalis*), 3 km for *Bombus pascuorum sensu lato*, 5 km for *B. lapidarius sensu lato*, 2 km for *B. pratorum* , and 10 km for *B. terrestris sensu lato* (based on Kraus et al. 2009; Lepais et al. 2010). These distances approximate queen dispersal and define the accessible area (M) from which pseudo‐absences were sampled (Phillips et al. 2009; Ranković Perišić et al. 2024).

Within each accessible area, we randomly sampled two background points per presence record, excluding occupied grid cells and masking out unsuitable habitats such as ocean and high mountain areas, after which we compiled environmental predictors at 1‐km^2^ resolution from WorldClim and Geodata and cropped them to each study region of the species to ensure that modeling occurred within ecologically relevant and accessible environments. We selected predictors known to influence bumblebee distributions (Torvanger et al. 2025): mean annual temperature, annual precipitation, elevation, and land cover. To reduce multicollinearity among predictors, we conducted pairwise Pearson correlation analyses and excluded highly correlated (|*r*| ≥ 0.7) variables, following recommendations for ecological modeling and species distribution analyses (Dormann et al. 2013).

### 2.2 Species Distribution Modeling and Habitat Suitability Analysis

Field surveys for any region are rarely spatially comprehensive or exhaustive across large geographic regions, which results in occurrence datasets that may contain substantial spatial bias. Consequently, raw distributional records alone may not reliably characterize a species' potential geographic distributions or habitat suitability pattern. Instead, geographic ranges must be modeled, typically by means of species distribution models (SDM) or ecological niche models (ENM). Mechanics that undergird SDM or ENM are the same; they differ in terms of emphasis, whether chiefly geographical or ecological (Peterson et al. 2011). Our focus was spatial, so we modeled species distributions (SDM) by means of boosted regression trees (BRTs), a machine‐learning algorithm well suited to capture nonlinear ecological relationships and variable interactions (Elith et al. 2008). To optimize model performance, we tuned hyperparameters and partitioned occurrence data using spatial blocking (method = “blocks,” *k* = 5), which reduces spatial autocorrelation and obtains unbiased estimates of predictive performance (Roberts et al. 2017). One block was withheld per iteration as an independent test set to evaluate model predictive accuracy. Model performance was assessed using the area under the receiver operating characteristic curve (AUC), true skill statistic (TSS), Boyce index, deviance, sensitivity, and specificity metrics, with mean and standard deviation computed across folds to quantify model discrimination (Landis and Koch 1977; Swets 1988). All models showed high predictive accuracy (AUC > 0.85; TSS > 0.7; see Table S1).

To retain the full gradient of predicted habitat suitability, we used continuous probability outputs rather than threshold binary maps (presence ≥ 0.5, absence < 0.5) (Jiménez‐Valverde and Lobo 2007; Liu et al. 2013). We therefore retained the continuous probability surfaces to capture the full gradient of predicted suitability and more accurately reflect heterogeneity (Peterson et al. 2011). From these predictions, we implemented a probability‐weighted sampling approach, in which 5000 samples were drawn per species in proportion to their predicted suitability values. This method preserves the relative contribution of highly suitable habitats as it captures the entire environmental spectrum, thereby avoiding overrepresentation of marginal areas inherent in uniform random sampling. A comparison of the two approaches (Figure S1) confirmed that probability‐weighted sampling more accurately reflects the environmental conditions associated with core habitat suitability.

### 2.3 Quantifying Distribution Overlap

Spatial overlap was compared between *V. bombylans* color morphs and their respective *Bombus* model species using a principal component analysis (PCA)‐based framework implemented in the *ecospat* package (Broennimann et al. 2012). Standardized predictor variables from the weighted samples were combined, and species densities were projected into a shared PCA space (*R* = 100 grid resolution), for each model–mimic pair (*V. b. “plumata”*–*B. terrestris s.l*., *V. b. “bombylans”*–*B. lapidarius s.l*., and *V. b. “haemorrhoidalis”*–*B. pascuorum s.l*.). We plotted species' occupancy densities along PCA axes and generated bivariate ellipses representing 95% confidence regions for each predicted spatial envelope and quantified “niche”, in our case the predicted geographical distribution by estimating Schoener's *D* and Hellinger's *I* indices quantified the degree of spatial overlap, ranging from 0 (no overlap) to 1 (complete overlap) (Warren et al. 2008).

We performed niche equivalency and niche similarity tests (Broennimann et al. 2012) to determine whether observed niche overlap exceeded that expected by chance. The equivalency test evaluated the null hypothesis that the two species occupy identical environmental niches by comparing the observed Schoener's *D* value with a null distribution generated by randomly reassigning occurrences between species while preserving sample sizes. The similarity test evaluated the null hypothesis that the observed overlap did not exceed random expectation by comparing the observed *D* value with a null distribution generated by randomly relocating occurrences of one species within the environmental background of the other. We based both tests on 1000 permutations and calculated *p*‐values for Schoener's *D* and Hellinger's *I*. Significant similarity tests indicate greater niche overlap than expected by chance, whereas significant equivalency tests reject the hypothesis that the two species occupy identical environmental niches.

### 2.4 Niche Size Estimation

Although the objective was to quantify spatial overlap rather than infer niches per se, the available methods for comparing estimated distributions are rooted in ecological niche modelling. We, therefore, interprete these metrics as measures of distributional overlap. We estimated niche breadth and probabilistic overlap by means of the *nicheROVER* framework (Swanson et al. 2015) on the probability‐weighted environmental data. For each species, multivariate niche parameters (mean and covariance) were estimated using a normal–inverse–Wishart prior via the niw.post() function, based on 1000 Monte Carlo samples. The 95% credible niche region volume represented niche size, and directional overlap probabilities quantified asymmetrical niche sharing between species (see Figure S2). Pairwise overlap probabilities and 95% credible intervals were summarized across 10,000 posterior samples. Probabilistic overlaps were visualized using density plots and PCA‐space projections to illustrate niche extent and shared environmental space among model–mimic pairs.

### 2.5 Spatial Association Between Mimics and Model Species

To test whether geographic distributions of *V. bombylans* color morphs were associated with the distributions of their putative *Bombus* model species, we fitted generalized linear models (GLMs) with a binomial error distribution. We extracted predicted habitat suitability values for candidate *Bombus* species from the species distribution models developed previously in our study. We aligned raster predictions to a common grid and extracted suitability values at the geographic coordinates of hoverfly occurrence records. We restricted mimic occurrences to mainland Norway to match the spatial extent of the environmental predictors used in the SDMs. To represent available environmental conditions, background points were randomly sampled within spatial buffers surrounding hoverfly occurrence locations, representing the accessible environment for each morph. Predicted *Bombus* suitability values were extracted at these locations using the same raster stack. Hoverfly occurrences were coded as presence (1) and background points as absence (0). Candidate GLMs were fitted in which mimic occurrence was modeled as a function of predicted habitat suitability of potential *Bombus* model species. Pairwise correlations among predictors were examined prior to model fitting. Because predicted suitability for *Bombus terrestris* sensu lato was highly correlated with that of *Bombus pascuorum* sensu lato, these predictors were not included together in the same candidate model to avoid multicollinearity. Models were compared using Akaike's Information Criterion (AIC), and the model with the lowest AIC was selected as the best‐supported model for each hoverfly morph.

Model fit was evaluated using a pseudo‐$R^{2}$, calculated from the reduction in deviance between the intercept‐only (null) model and the fitted model. Thereafter the residual spatial autocorrelation was assessed using Moran's *I* based on a nearest‐neighbor spatial weighting matrix. Where substantial spatial autocorrelation was detected, spatial eigenvector mapping was explored (Dormann et al. 2007). Spatial eigenvectors were generated using the spdep package in R and added to the best‐supported biological model. However, inclusion of spatial eigenvectors altered model coefficients or model ranking only slightly; therefore, results are presented from the simpler GLM models without spatial eigenvector terms.

## 3 Results

**FIGURE 1:**
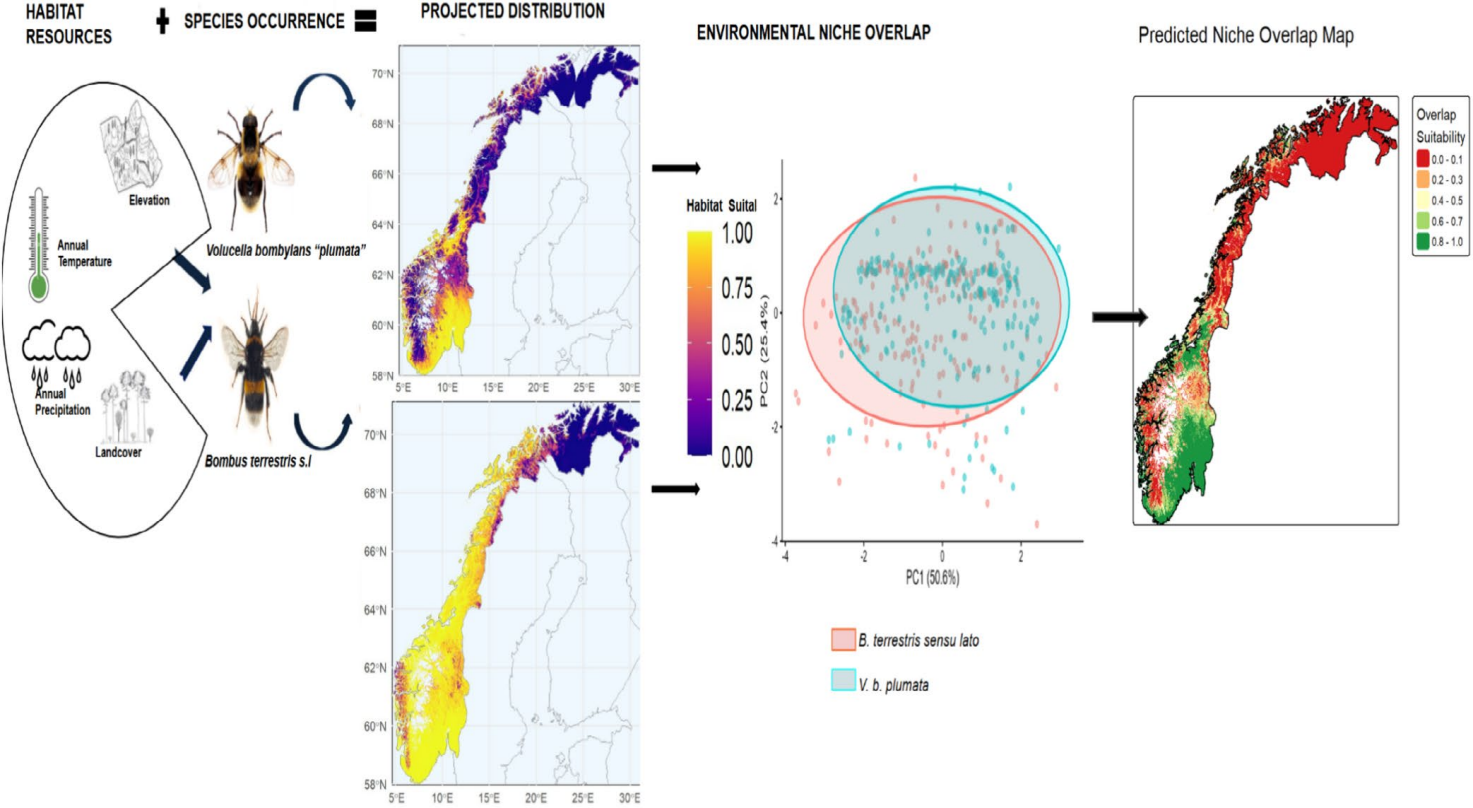
Geographic distribution overlap analysis framework: Modeled geographic distributions and spatial overlap among *Bombus terrestris* *s.l*. and corresponding *Volucella bombylans “plumata”*.

**FIGURE 2:**
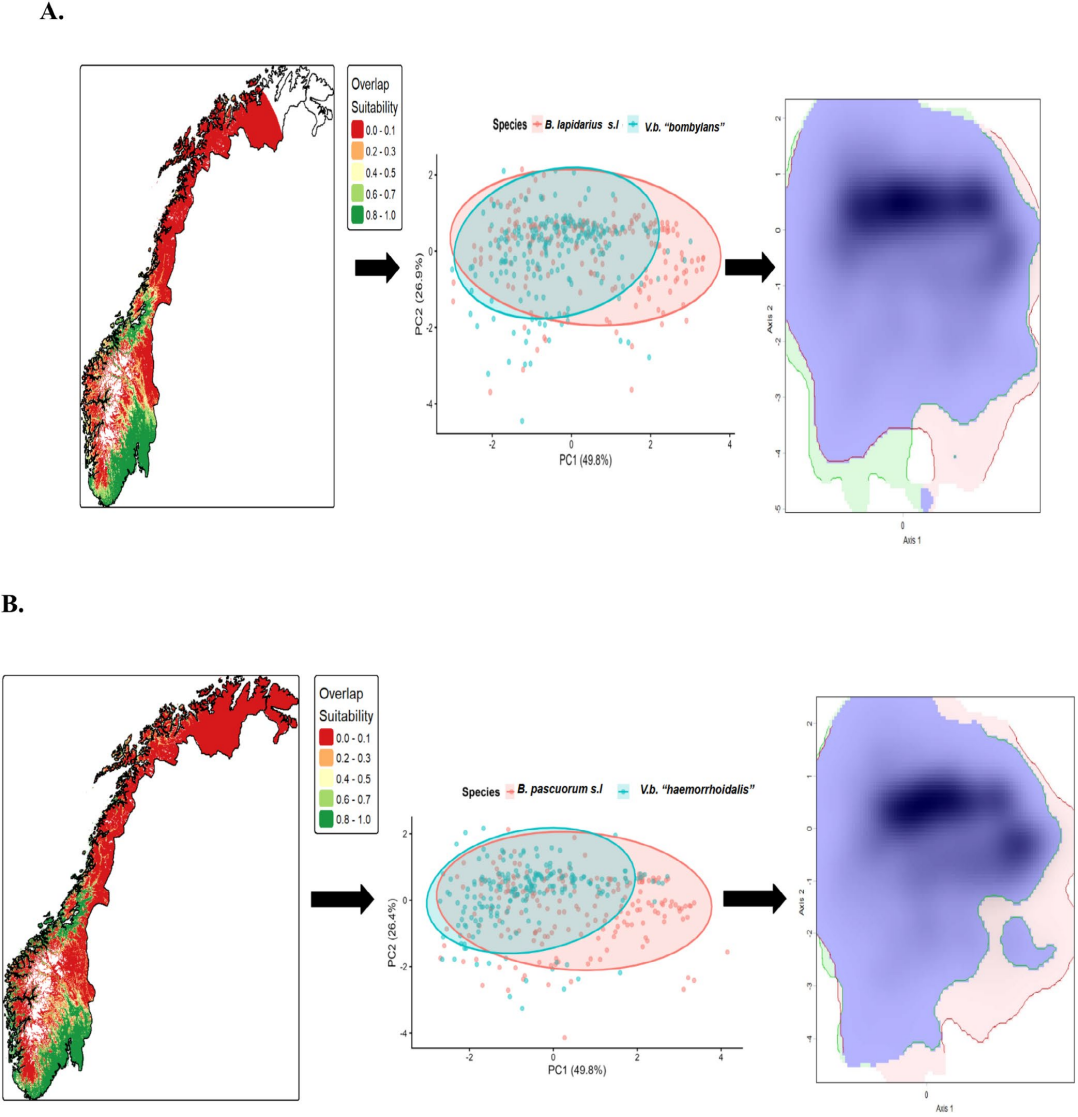
Distributional overlap between the color morphs of *Volucella bombylans* and their putative *Bombus* model species in geographic and environmental space. Shown are (A) *V. b. bombylans* and *Bombus lapidarius sensu lato* and (B) *V. b. “haemorrhoidalis”* and *Bombus pascuorum sensu lato*. The left panels show the geographic overlap in predicted habitat suitability across Norway, with colors indicating increasing overlap from low (red) to high (green). The middle panels show species occurrences projected onto the first two principal components (PC1 and PC2) summarizing environmental variation, with ellipses representing the environmental niche occupied by each species. The right panels show kernel density estimates of environmental occupancy in PCA‐transformed environmental space. Darker blue shading indicates regions of highest occurrence density (core environmental niche), whereas lighter shading indicates less frequently occupied environmental conditions. Red and green contour lines delineate the environmental niche of each species, and their overlap illustrates the degree of environmental niche similarity between model and mimic species. The overlap map represents the overlap between the predicted habitat suitability of *V. b. “bombylans”* and *Bombus lapidarius* *s.l*. The smaller predicted extent of *V. b. “bombylans”* reflects its narrower predicted distribution (within its accessible area) relative to its putative model.

Annual mean temperature was the main environmental factor influencing the distributions of all *Bombus* species and *V. bombylans* morphs, although the importance of other variables differed among taxa. Precipitation strongly influenced *V. b. “plumata”* and *V. b. “bombylans”*, while elevation was more important for *V. b. “haemorrhoidalis”*. Overall, the environmental drivers affecting the hoverfly morphs were broadly similar to those shaping the distributions of their corresponding *Bombus* models, suggesting that mimic–model pairs share similar climatic requirements. Prediction maps (Figures 1 and 2; Figure S3) showed species‐specific responses to environmental gradients and provided context for patterns of geographic overlap. Distributional overlap was strongest between *Bombus terrestris* *s.l*. and *V. b. “plumata”*, and between *B. lapidarius* *s.l*. and *V. b. “bombylans”*, with both pairs showing statistically significant equivalency and similarity (*p* < 0.01) (see Table 1). In contrast, *B. pascuorum s.l*. and *V. b. “haemorrhoidalis”* exhibited moderate overlap, and neither equivalency nor similarity tests were significant, suggesting that these species occupy partially distinct regions of a shared niche space.

**TABLE 1:** Distributional overlap metrics and statistical tests of equivalency and similarity between Bumblebee models and their Hoverfly mimics.

Model Bumblebee species/mimic hoverfly species	Schoener's *D* index	Hellinger's *I* index	Niche equivalency *p*	Niche similarity test *p*
*B. terrestris s. l./V. b. “plumata”*	0.90	0.94	0.009	0.02
*B. lapidarius s.l./V. b. “bombylans”*	0.77	0.85	0.01	0.03
*B. pascuorum s. l./V. b. “haemorrhoidalis”*	0.53	0.73	0.17	0.07

Niche‐nesting analyses showed that *B. lapidarius* *s.l*. occupied a subset of the broader niches of *B. pascuorum* *s.l*. and *B. terrestris* *s.l*. Likewise, *V. b. “plumata”* encompassed the environmental niches of all other *V. bombylans* morphs (Figure S4), demonstrating substantial climatic niche nestedness and overlap rather than distinct environmental segregation (Tables S2 and S3). Directional overlap was greatest between *B. terrestris* *s.l*. and *V. b. “plumata”* (mean ≈81%, 95% credible interval: 82.5%–88.6%) and lowest between *B. pascuorum* *s.l*. and *V. b. “haemorrhoidalis”* (~62%), suggesting a gradient of niche convergence across mimic–model pairs (Figure S2).

To determine whether *V. bombylans* morphs were associated more strongly with their putative *Bombus* models than with alternative *Bombus* species, we compared how hoverfly occurrence varied with the habitat suitability of different *Bombus* species. For *V. b. “bombylans” and V. b. “plumata”*, the putative model species received support (Table 2). However, alternative candidate models also received comparable support (ΔAIC < 2), suggesting that multiple *Bombus* species explained hoverfly occurrence equally well. In contrast, the intercept‐only (null) model received the greatest support for *V. b. “haemorrhoidalis”*, indicating that models including habitat suitability of candidate *Bombus* species received no greater support than the null model.

**TABLE 2:** Model selection statistics (AIC and ΔAIC) for generalized linear models evaluating associations between *V. bombylans* morphs and candidate *Bombus* species.

Morph	Occurrences (*n*)	Candidate model	AIC	ΔAIC
*V. b. “plumata”*	654	***B. lapidarius***	**1391.05**	**0.00**
		*B. lapidarius* *+* *B. pascuorum* ^†^	1392.76	1.71
		*B. lapidarius* *+* *B. terrestris* ^†^	1392.97	1.92
		Full model	1393.49	2.43
		*B. terrestris (putative model)*	1427.30	36.25
		*B. pascuorum*	1437.05	46.00
		*Null model (intercept only)*	1441.29	50.23
*V. b. “bombylans”*	198	***B. lapidarius (putative model)***	**474.87**	**0.00**
		*B. lapidarius* *+* *B. terrestris* ^†^	476.41	1.54
		*B. lapidarius* *+* *B. pascuorum* ^†^	476.48	1.61
		Full model	478.27	3.40
		*B. terrestris*	478.94	4.07
		*B. pascuorum*	481.75	6.88
		*Null model (intercept only)*	481.77	6.90
*V. b. “haemorrhoidalis”*	155	***Null model (intercept only)***	**356.64**	**0.00**
		*B. lapidarius*	357.85	1.21
		*B. terrestris*	358.35	1.71
		*B. pascuorum (putative model)*	358.61	1.97
		*B. lapidarius* *+* *B. pascuorum*	359.66	3.02
		*B. lapidarius* *+* *B. terrestris*	359.84	3.20
		Full model	361.50	4.86

*Note:* The highest‐ranked models (ΔAIC = 0) are shown in bold. ΔAIC represents the difference in AIC between each model and the top‐ranked model within each candidate set. Models marked with † received comparable AIC support (ΔAIC < 2), but the additional predictor (*B. terrestris s.l* or *B. pascuorum s.l*) showed no independent association with hoverfly occurrence when included alongside *B. lapidarius s.l* (see Table S4). Sample sizes (*n*) indicate the number of hoverfly occurrence records used in each analysis.

**FIGURE 3:**
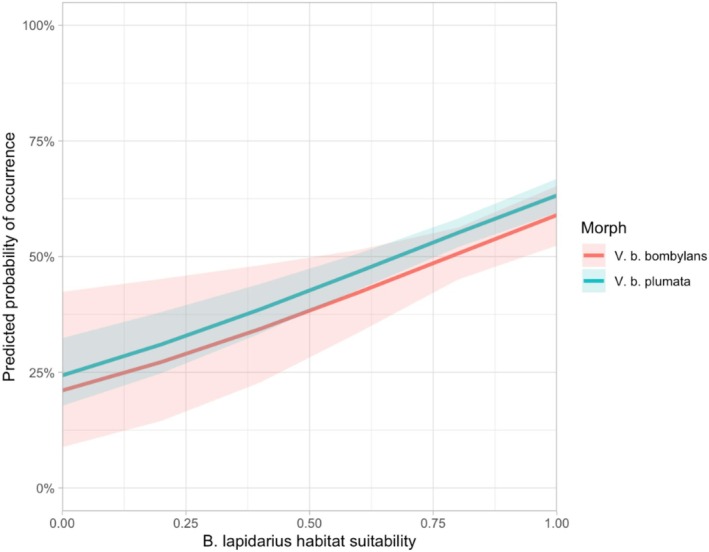
Predicted occurrence probability of *Volucella bombylans* morphs across the *Bombus lapidarius* habitat suitability gradient. Lines show predicted probabilities from generalized linear models relating hoverfly occurrence to predicted habitat suitability for *B. lapidarius* *s.l*. Shaded areas represent 95% confidence intervals.

Within the highest‐ranked models, the occurrence of *V. b. “plumata”* and *V. b. “bombylans”* increased with increasing *B. lapidarius* habitat suitability (Table 3; Figure 3). Although two additional candidate models for *V. b. “plumata”* received comparable AIC support (ΔAIC < 2), neither *B. terrestris* *s.l*. nor *B. pascuorum s.l* showed evidence of an independent association when included alongside *B. lapidarius* (Table S4), indicating that these additional predictors contributed little explanatory power beyond that provided by *B. lapidarius* .

**TABLE 3:** Parameter estimates from the best‐supported generalized linear models relating occurrence of *V. bombylans* color morphs to *B. lapidarius* habitat suitability.

Morph	Predictor	β ± SE	*z*	Wald *χ* ^2^	*p*
*V. b. “plumata”*	*B. lapidarius s.l*	1.68 ± 0.24	6.94	48.20	< 0.001
*V. b. “bombylans”*	*B. lapidarius s.l*	1.68 ± 0.59	2.87	8.24	0.004
*V. b. “haemorrhoidalis”*	—	—	—	—	—

*Note:* Parameter estimates and Wald statistics are not reported for *V. b. “haemorrhoidalis”* because the intercept‐only model was the best‐supported model.

## 4 Discussion

We found strong geographic overlap between *V. bombylans* color morphs and their corresponding *Bombus* model species, which supports the central prediction of Batesian mimicry that effective mimicry requires some degree of spatial co‐occurrence between mimics and models (Bates 1862; Ruxton 2010). Although some mimic–model pairs did not completely overlap across their distributions, mimics still occurred within portions of the broader niche breadth of their presumed models. This pattern aligns with theoretical expectations that Batesian mimics need not occur entirely within the range of their models, provided some degree of sympatry permits predator learning and reinforcement of warning signals (Pfennig and Mullen 2010; Ruxton 2010). Because Bombus species are only mildly unpalatable, effective mimicry in the *Bombus*–*Volucella* system likely depends more strongly on repeated co‐occurrence and predator learning than mimicry systems involving highly defended models (Sherratt 2002; Barnett et al. 2007). Overlap strength varied among morphs, with *V. b. plumata* and *B. terrestris* *s.l*. showing the strongest niche similarity and *V. b. “haemorrhoidalis”* and *B. pascuorum* *s.l*. the weakest. Variation in model defensiveness may contribute to this pattern because highly defensive species such as *B. terrestris* and *B. lucorum* are more memorable to predators than comparatively docile species such as *B. pascuorum* (Alford 1975; Rupp 1989; Kearns and Thomson 2001). Differences in phenology may also contribute because *B. terrestris* emerges earlier in the season than *B. lapidarius* and *B. pascuorum* (Kells and Goulson 2003; Blasi et al. 2023), potentially reducing opportunities for co‐occurrence between later‐emerging mimic–model pairs, although we did not explicitly test the effects of defenses or phenology.

Consistent with this pattern, *V. b. “haemorrhoidalis”* showed the weakest overlap with its presumed model, with nearly 40% of its predicted niche occurring outside that of *B. pascuorum*. Several Bombus species included within the *B. pascuorum* color‐space grouping used in our analyses (e.g., *B. humilis*, *B. consobrinus*, *B. hypnorum*, and *B. cingulatus*), occupy more restricted boreal or alpine distributions (Kristiansen 2006; Rasmont et al. 2015; Rasmont and Iserbyt 2010) which potentially reduce the breadth and local abundance of closely resembling models available to this mimic morph across much of the landscape. Also, fine‐scale genomic differentiation and associated color variation within *B. pascuorum* populations even within the Norwegian population may further alter the range of locally available model phenotypes across regions (Lecocq et al. 2015) Despite this weak overlap, *V. b. “haemorrhoidalis”* reaches high local abundance in parts of France despite Bombus communities similar to those in the United Kingdom (Edmunds and Reader 2014) suggesting that local ecological conditions influence mimic–model associations. Its persistence may also reflect frequency‐dependent predator behavior, where predators disproportionately attack common prey forms and thereby confer a rarity advantage on uncommon mimics (Finkbeiner et al. 2018).

Despite differences among expected mimic–model pairs, hoverfly morphs did not strictly track presumed *Bombus* model species. Instead, *B. lapidarius* habitat suitability best predicted occurrence across *V. bombylans* morphs, including morphs traditionally associated with *B. terrestris* . Our analyses revealed a nested pattern of climatic niches among *Bombus* models, with *B. terrestris* *s.l*. and *B. pascuorum* *s.l*. occupying broader niche space, while *B. lapidarius* *s.l*. occupied a narrower nested subset. This pattern likely reflects differences in climatic tolerance breadth rather than climatic optima alone. Because *B. lapidarius* is one of the more thermophilic European bumblebee species associated with warmer habitats (Goulson 2009; Rasmont et al. 2015) its distribution may represent a restricted warm‐adapted subset within the broader climatic ranges of *B. terrestris* and *B. pascuorum* . This pattern suggests that *B. lapidarius* occupies environmental space shared across multiple *Bombus* assemblages, potentially capturing climatic conditions common to both broader and narrower model niches. The broad climatic niche of the *B. terrestris* complex likely reflects the inclusion of cold‐adapted taxa such as *B. lucorum* , which commonly occur in cooler, wetter, and more forested habitats at higher elevations (Geue and Thomassen 2020). Common *Bombus* species such as *B. terrestris* , *B. lucorum* , and *B. lapidarius* frequently co‐occur across similar habitats despite broad overlap in resource use (Goulson et al. 2008; Goulson and Darvill 2004), potentially exposing predators to shared *Bombus* warning signals across similar environments. Predators may therefore generalize among co‐occurring warning patterns rather than respond to individual model species (Exnerová et al. 2007; Kazemi et al. 2014). Such predator generalization may reduce selection for precise resemblance to a single defended species, allowing mimics to gain protection from multiple co‐occurring models (Ruxton et al. 2019). Similar patterns have been documented in Batesian mimicry systems involving multiple model species (Akcali et al. 2019). Spatial heterogeneity in *Bombus* communities may further weaken strict pairwise associations and maintain polymorphism across shared predator environments (Joron and Iwasa 2005). Our results therefore suggest that predator‐mediated selection in *V. bombylans* operates across shared *Bombus*‐associated environments rather than through strict one‐to‐one mimic–model relationships.

Overall, model–mimic pairs generally responded similarly to environmental gradients, with both Bombus species and corresponding *V. bombylans* morphs increasing with temperature and decreasing with precipitation. Similar climatic associations have been reported in other Bombus species and hoverflies (Ball and Morris 2021; Lu and Huang 2023; López‐Aguilar et al. 2024). The main exception involved the *B. pascuorum* –*V. b. “haemorrhoidalis”* pair, where the model showed stronger associations with wetter habitats. This divergence likely reflects ecological differences because *B. pascuorum* commonly nests in humid, tussock‐rich habitats (Svensson et al. 2000; Kells and Goulson 2003).

We found few occurrence records for rare *V. bombylans* color morphs, consistent with earlier field observations that some morphs occur at very low frequencies (Edmunds and Reader 2014). Despite the limited number of occurrence records, our models performed well. Even so, small sample sizes and the inherent biases of citizen science records, such as uneven sampling effort and presence‐only observations, remain key limitations (Burgess et al. 2017; Shirey et al. 2023). These limitations also restricted our ability to account for fine‐scale ecological processes that likely influence mimic–model associations, including local predator behavior, species interactions, and phenological overlap. Future studies investigating mimic–model spatial dynamics should therefore incorporate species traits, biotic interactions, and hierarchical joint‐species distribution approaches to better resolve how ecological communities structure polymorphic mimicry across space and time.

## Author Contributions

**Blessing Chidinma Umeh:** conceptualization (equal), data curation (lead), formal analysis (lead), investigation (equal), writing – original draft (lead). **Markus A. K. Sydenham:** funding acquisition (equal), methodology (equal), supervision (equal), writing – review and editing (equal). **Michael A. Patten:** conceptualization (equal), funding acquisition (equal), supervision (equal), writing – review and editing (equal).

## Funding

This research was supported by a 3‐year grant from the Norwegian Ministry of Education and Research.

## Conflicts of Interest

The authors declare no conflicts of interest.

## Supporting information


**Table S1:** Model evaluation metrics for seven species distribution models using test data. Average and standard deviation (SD) are shown for threshold, AUC, sensitivity, specificity, deviance, Boyce index, and True Skill Statistic (TSS). All models performed well (AUC > 0.85, TSS > 0.7), indicating good discrimination between suitable and unsuitable habitat.
**Table S2:** Environmental niche overlap metrics between Bumblebee models.
**Table S3:** Environmental niche overlap metrics between also for hoverfly mimics.
**Table S4:** Parameter estimates for competing candidate models (ΔAIC < 2).
**Figure S1:** Compare weighted sampling vs. random sampling of the habitat suitability predictions.
**Figure S2:** Niche size of species pairs: Posterior distributions of directional ecological niche overlap between bumblebee model species and their respective hoverfly mimics. The plots show the estimated probability that each mimic's niche falls within the niche region of its corresponding model, based on 1000 posterior samples using the nicheROVER framework. (A) *Bombus terrestris sensu latos and Volucella bombylans plumata*; (B) *Bombus lapidarius and Volucella bombylans bombylans* and (C) *Bombus pascuorum sensu latos and Volucella bombylans haemorrhoidalis*. Shaded histograms represent the distribution of overlap probabilities, with vertical lines denoting the mean (solid) and 95% credible intervals (dashed).
**Figure S3:** Predicted habitat suitability for (A) *Volucella bombylans plumata* (top) and *Bombus terrestris s.l*. (bottom) (B) *Volucella bombylans bombylans* (top) and *Bombus lapidarius s.l*. (bottom) (C) *Volucella bombylans haemorrhoidalis* (top) and *Bombus pascuorum* *s.l*. (bottom) across Norway.
**Figure S4:** (Niche overlap PCA plots). Environmental niche overlap among *Volucella bombylans* color morphs based on PCA of environmental space: (A) *V. b. plumata* vs. *V. b. bombylans*, (B) *V. b. plumata* vs. *V. b. haemorrhoidalis*, and (C) *V. b. bombylans* vs. *V. b. haemorrhoidalis*.

## Data Availability

All data supporting this study are publicly available on Zenodo at https://doi.org/10.5281/zenodo.18411221.

## References

[ece374146-bib-0001] Aiello‐Lammens, M. E. , R. A. Boria , A. Radosavljevic , B. Vilela , and R. P. Anderson . 2015. “spThin: An R Package for Spatial Thinning of Species Occurrence Records for Use in Ecological Niche Models.” Ecography 38: 541–545.

[ece374146-bib-0002] Akcali, C. K. , H. A. Pérez‐Mendoza , D. W. Kikuchi , and D. W. Pfennig . 2019. “Multiple Models Generate a Geographical Mosaic of Resemblance in a Batesian Mimicry Complex.” Proceedings of the Royal Society B: Biological Sciences 286: 1–9. 10.1098/rspb.2019.1519 PMC6784714 31530146

[ece374146-bib-0003] Akcali, C. K. , and D. W. Pfennig . 2014. “Rapid Evolution of Mimicry Following Local Model Extinction.” Biology Letters 10: 20140304. 24919704 10.1098/rsbl.2014.0304 PMC4090552

[ece374146-bib-0004] Akcali, C. K. , and D. W. Pfennig . 2017. “Geographic Variation in Mimetic Precision Among Different Species of Coral Snake Mimics.” Journal of Evolutionary Biology 30: 1420–1428. 28425157 10.1111/jeb.13094

[ece374146-bib-0005] Alford, D. V. 1975. Bumblebees of London. Davis‐Poynter Ltd.

[ece374146-bib-0006] Ball, S. , and R. Morris . 2021. “Recent Range Expansion in British Hoverflies (Diptera, Syrphidae).” Dipterists Digest 28: 59–87.

[ece374146-bib-0007] Barnett, C. A. , M. Bateson , and C. Rowe . 2007. “State‐Dependent Decision Making: Educated Predators Strategically Trade Off the Costs and Benefits of Consuming Aposematic Prey.” Behavioral Ecology 18: 645–651.

[ece374146-bib-0008] Bates, H. W. 1862. “Contributions to an Insect Fauna of the Amazon Valley (Lepidoptera: Heliconidae).” Transactions of the Linnean Society of London 6: 73–77.

[ece374146-bib-0009] Blasi, M. , R. Carrié , C. Fägerström , E. Svensson , and A. S. Persson . 2023. “Historical and Citizen‐Reported Data Show Shifts in Bumblebee Phenology Over the Last Century in Sweden.” Biodiversity and Conservation 32: 1523–1547.

[ece374146-bib-0010] Bond, A. B. 2007. “The Evolution of Color Polymorphism: Crypticity, Searching Images, and Apostatic Selection.” Annual Review of Ecology, Evolution, and Systematics 38: 489–514.

[ece374146-bib-0011] Broennimann, O. , M. C. Fitzpatrick , P. B. Pearman , et al. 2012. “Measuring Ecological Niche Overlap From Occurrence and Spatial Environmental Data.” Global Ecology and Biogeography 21: 481–497.

[ece374146-bib-0076] Brower, J. V. Z. 1960. “Experimental Studies of Mimicry. IV. The Reactions of Starlings to Different Proportions of Models and Mimics.” American Naturalist 94, no. 877: 271–282.

[ece374146-bib-0012] Burgess, H. K. , L. B. DeBey , H. E. Froehlich , et al. 2017. “The Science of Citizen Science: Exploring Barriers to Use as a Primary Research Tool.” Biological Conservation 208: 113–120.

[ece374146-bib-0013] Dittrich, W. , F. Gilbert , P. Green , P. Mcgregor , and D. Grewcock . 1993. “Imperfect Mimicry: A Pigeon's Perspective.” Proceedings of the Royal Society B: Biological Sciences 251: 195–200.

[ece374146-bib-0014] Dormann, C. F. , J. Elith , S. Bacher , et al. 2013. “Collinearity: A Review of Methods to Deal With It and a Simulation Study Evaluating Their Performance.” Ecography 36: 27–46.

[ece374146-bib-0015] Dormann, C. F. , J. M. Mcpherson , M. B. Arau , et al. 2007. “Methods to Account for Spatial Autocorrelation in the Analysis of Species Distributional Data: A Review.” Ecography 30: 609–628.

[ece374146-bib-0016] Edmunds, M. 2000. “Why Are There Good and Poor Mimics?” Biological Journal of the Linnean Society 70: 459–466.

[ece374146-bib-0017] Edmunds, M. 2008. “Hoverflies: The Garden Mimics.” Biologist 55: 202–207.

[ece374146-bib-0018] Edmunds, M. , and T. Reader . 2014. “Evidence for Batesian Mimicry in a Polymorphic Hoverfly.” Evolution 68: 827–839. 24410141 10.1111/evo.12308

[ece374146-bib-0019] Elith, J. , J. R. Leathwick , and T. Hastie . 2008. “A Working Guide to Boosted Regression Trees.” Journal of Animal Ecology 77: 802–813. 18397250 10.1111/j.1365-2656.2008.01390.x

[ece374146-bib-0020] Exnerová, A. , P. Štys , E. Fučíková , et al. 2007. “Avoidance of Aposematic Prey in European Tits (Paridae): Learned or Innate?” Behavioral Ecology 18: 148–156.

[ece374146-bib-0021] Fick, S. E. , and R. J. Hijmans . 2017. “WorldClim 2: New 1‐Km Spatial Resolution Climate Surfaces for Global Land Areas.” International Journal of Climatology 37: 4302–4315.

[ece374146-bib-0077] Finkbeiner, S. D. , P. A. Salazar , S. Nogales , et al. 2018. “Frequency Dependence Shapes the Adaptive Landscape of Imperfect Batesian Mimicry.” Proceedings of the Royal Society B: Biological Sciences 285, no. 1876: 20172786. 10.1098/rspb.2017.2786 PMC5904311 29618547

[ece374146-bib-0022] Geue, J. C. , and H. A. Thomassen . 2020. “Unraveling the Habitat Preferences of Two Closely Related Bumble Bee Species in Eastern Europe.” Ecology and Evolution 10: 4773–4790. 32551060 10.1002/ece3.6232 PMC7297791

[ece374146-bib-0023] Goulson, D. 2009. Bumblebees: Behaviour, Ecology, and Conservation. Oxford University Press.

[ece374146-bib-0024] Goulson, D. , and B. Darvill . 2004. “Niche Overlap and Diet Breadth in Bumblebees; Are Rare Species More Specialized in Their Choice of Flowers?” Apidologie 35: 55–63.

[ece374146-bib-0025] Goulson, D. , G. C. Lye , and B. Darvill . 2008. “Diet Breadth, Coexistence and Rarity in Bumblebees.” Biodiversity and Conservation 17: 3269–3288.

[ece374146-bib-0026] Harper, G. R. , and D. W. Pfennig . 2007. “Mimicry on the Edge: Why Do Mimics Vary in Resemblance to Their Model in Different Parts of Their Geographical Range?” Proceedings. Biological Sciences 274: 1955–1961. 17567563 10.1098/rspb.2007.0558 PMC2275182

[ece374146-bib-0027] Howarth, B. , C. Clee , and M. Edmunds . 2000. “The Mimicry Between British Syrphidae (Diptera) and Aculeate Hymenoptera.” British Journal of Entomology and Natural History 13: 1–39.

[ece374146-bib-0028] Howarth, B. , and M. Edmunds . 2000. “The Phenology of Syrphidae (Diptera): Are They Batesian Mimics of Hymenoptera?” Biological Journal of the Linnean Society 71: 437–457.

[ece374146-bib-0029] Howarth, B. , M. Edmunds , and F. Gilbert . 2016. “Does the Abundance of Hoverfly (Syrphidae) Mimics Depend on the Numbers of Their Hymenopteran Models?” Evolution 58: 367–375. 15068353

[ece374146-bib-0030] Iserbyt, A. , J. Bots , S. van Dongen , J. J. Ting , H. van Gossum , and T. N. Sherratt . 2011. “Frequency‐Dependent Variation in Mimetic Fidelity in an Intraspecific Mimicry System.” Proceedings of the Royal Society B: Biological Sciences 278: 3116–3122. 10.1098/rspb.2011.0126 PMC3158940 21367784

[ece374146-bib-0031] Jiménez‐Valverde, A. , and J. M. Lobo . 2007. “Threshold Criteria for Conversion of Probability of Species Presence to Either‐ or Presence‐Absence.” Acta Oecologica 31: 361–369.

[ece374146-bib-0079] Joron, M. , and Y. Iwasa . 2005. “The Evolution of a Müllerian Mimic in a Spatially Distributed Community.” Journal of Theoretical Biology 237, no. 1: 87–103. 15975598 10.1016/j.jtbi.2005.04.005

[ece374146-bib-0032] Kazemi, B. , G. Gamberale‐Stille , B. Tullberg , and O. Leimar . 2014. “Stimulus Salience as an Explanation for Imperfect Mimicry.” Current Biology 24: 965–969. 24726157 10.1016/j.cub.2014.02.061

[ece374146-bib-0033] Kearns, C. A. , and J. D. Thomson . 2001. The Natural History of Bumblebees: A Sourcebook for Investigations. University Press of Colorado.

[ece374146-bib-0034] Kells, A. R. , and D. Goulson . 2003. “Preferred Nesting Sites of Bumblebee Queens (Hymenoptera: Apidae) in Agroecosystems in the UK.” Biological Conservation 109: 165–174.

[ece374146-bib-0035] Kokko, H. , J. Mappes , and L. Lindström . 2003. “Alternative Prey Can Change Model‐Mimic Dynamics Between Parasitism and Mutualism.” Ecology Letters 6: 1068–1076.

[ece374146-bib-0036] Kraus, F. B. , S. Wolf , and R. F. A. Moritz . 2009. “Male Flight Distance and Population Substructure in the Bumblebee *Bombus terrestris* .” Journal of Animal Ecology 78: 247–252. 19120605 10.1111/j.1365-2656.2008.01479.x

[ece374146-bib-0037] Kristiansen, D. 2006. Foraging Activity of Bumblebees (Bombus) in Relation to Flower Resources on Arable Land: A Follow‐Up 13 Years Later. Norwegian University of Life Sciences.

[ece374146-bib-0038] Kristiansen, E. B. , S. D. Finkbeiner , R. I. Hill , L. Prusa , and S. P. Mullen . 2018. “Testing the Adaptive Hypothesis of Batesian Mimicry Among Hybridizing North American Admiral Butterflies.” Evolution 72: 1436–1448. 10.1111/evo.13488 29851081

[ece374146-bib-0039] Landis, J. R. , and G. G. Koch . 1977. “The Measurement of Observer Agreement for Categorical Data.” Biometrics 33: 159–174. 843571

[ece374146-bib-0040] Lecocq, T. , N. Brasero , B. Martinet , I. Valterovà , and P. Rasmont . 2015. “Highly Polytypic Taxon Complex: Interspecific and Intraspecific Integrative Taxonomic Assessment of the Widespread Pollinator *Bombus pascuorum* Scopoli 1763 (Hymenoptera: Apidae).” Systematic Entomology 40: 881–890.

[ece374146-bib-0041] Lepais, O. , B. Darvill , S. O'Connor , et al. 2010. “Estimation of Bumblebee Queen Dispersal Distances Using Sibship Reconstruction Method.” Molecular Ecology 19: 819–831. 20089127 10.1111/j.1365-294X.2009.04500.x

[ece374146-bib-0042] Lindström, L. , R. V. Alatalo , A. Lyytinen , and J. Mappes . 2004. “The Effect of Alternative Prey on the Dynamics of Imperfect Batesian and Müllerian Mimicries.” Evolution 58: 1294–1302. 15266978 10.1111/j.0014-3820.2004.tb01708.x

[ece374146-bib-0043] Liu, C. , M. White , and G. Newell . 2013. “Selecting Thresholds for the Prediction of Species Occurrence With Presence‐Only Data.” Journal of Biogeography 40: 778–789.

[ece374146-bib-0044] López‐Aguilar, T. P. , J. Montalva , B. Vilela , et al. 2024. “Niche Analyses and the Potential Distribution of Four Invasive Bumblebees Worldwide.” Ecology and Evolution 14: 1–15. 10.1002/ece3.11200 PMC10985363 38571800

[ece374146-bib-0045] Lu, M. L. , and J. Y. Huang . 2023. “Predicting Negative Effects of Climate Change on Taiwan's Endemic Bumblebee *Bombus formosellus* .” Journal of Insect Conservation 27: 193–203.

[ece374146-bib-0046] Mallet, J. , M. Joro , M. James , and M. Joron . 1999. “Evolution of Diversity in Warning Color and Mimicry: Polymorphisms, Shifting Balance, and Speciation.” Annual Review of Ecology and Systematics 30: 201–233.

[ece374146-bib-0047] Mappes, J. , N. Marples , and J. A. Endler . 2005. “The Complex Business of Survival by Aposematism.” Trends in Ecology & Evolution 20: 598–603. 16701442 10.1016/j.tree.2005.07.011

[ece374146-bib-0048] Mostler, G. 1934. “Observations on the Question of Wasp Mimicry.” Journal of Animal Morphology and Ecology 29: 381–454.

[ece374146-bib-0049] Penney, H. , C. Hassall , J. Skevington , K. R. Abbott , and T. Sherratt . 2012. “A Comparative Analysis of the Evolution of Imperfect Mimicry.” Nature 483: 461–464. 22437614 10.1038/nature10961

[ece374146-bib-0050] Peterson, A. T. , J. Soberón , R. G. Pearson , et al. 2011. “Ecological Niches and Geographic Distributions.” In Ecological Niches and Geographic Distributions. Princeton University Press.

[ece374146-bib-0051] Pfennig, D. W. , W. R. Harcombe , and K. S. Pfennig . 2001. “Frequency‐Dependent Batesian Mimicry.” Nature 410: 323. 11268195 10.1038/35066628

[ece374146-bib-0052] Pfennig, D. W. , G. R. Harper , A. F. Brumo , W. R. Harcombe , and K. S. Pfennig . 2007. “Population Differences in Predation on Batesian Mimics in Allopatry With Their Model: Selection Against Mimics Is Strongest When They Are Common.” Behavioral Ecology and Sociobiology 61: 505–511.

[ece374146-bib-0053] Pfennig, D. W. , and S. P. Mullen . 2010. “Mimics Without Models: Causes and Consequences of Allopatry in Batesian Mimicry Complexes.” Proceedings of the Royal Society B: Biological Sciences 277: 2577–2585. 10.1098/rspb.2010.0586 PMC2982051 20484238

[ece374146-bib-0054] Phillips, S. J. , M. Dudík , J. Elith , et al. 2009. “Sample Selection Bias and Presence‐Only Distribution Models: Implications for Background and pseudo‐Absence Data.” Ecological Applications 19: 181–197. 19323182 10.1890/07-2153.1

[ece374146-bib-0055] Prudic, K. L. , and J. C. Oliver . 2008. “Once a Batesian Mimic, Not Always a Batesian Mimic: Mimic Reverts Back to Ancestral Phenotype When the Model Is Absent.” Proceedings of the Royal Society B: Biological Sciences 275: 1125–1132. 10.1098/rspb.2007.1766 PMC2602694 18285285

[ece374146-bib-0056] Ranković Perišić, M. , T. Nikolić Lugonja , S. Radenković , et al. 2024. “Global Warming—Friend or Enemy of Hoverflies (Diptera: Syrphidae) in Montenegro.” Journal of Insect Conservation 28: 1223–1245.

[ece374146-bib-0057] Rasmont, P. , M. Franzén , T. Lecocq , et al. 2015. Climatic Risk and Distribution Atlas of European Bumblebees. Pensoft Publishers.

[ece374146-bib-0058] Rasmont, P. , and S. Iserbyt . 2010. Atlas of the European Bees: Genus Bombus. 3rd ed. STEP Project. Atlas Hymenoptera.

[ece374146-bib-0059] Ries, L. , and S. P. Mullen . 2008. “A Rare Model Limits the Distribution of Its More Common Mimic: A Twist on Frequency‐Dependent Batesian Mimicry.” Evolution 62: 1798–1803. 18410533 10.1111/j.1558-5646.2008.00401.x

[ece374146-bib-0060] Roberts, D. R. , V. Bahn , S. Ciuti , et al. 2017. “Cross‐Validation Strategies for Data With Temporal, Spatial, Hierarchical, or Phylogenetic Structure.” Ecography 40: 913–929.

[ece374146-bib-0061] Rupp, L. 1989. “Die Mitteleuropäischen Arten der Gattung Volucelle (Diptera, Syrphidae) als Kommensalen und Parasitoide in den Nestern von Hummeln und sozialen Wespen: Untersuchungen zur Wirtsfindung, Larvalbiologie und Mimikry”.

[ece374146-bib-0062] Ruxton, G. D. 2010. “Defensive Coloration.” In Encyclopedia of Animal Behavior, edited by M. D. Breed and J. Moore , 487–492. Academic Press.

[ece374146-bib-0063] Ruxton, G. D. , W. L. Allen , T. N. Sherratt , and M. P. Speed . 2019. Avoiding Attack: The Evolutionary Ecology of Crypsis, Aposematism, and Mimicry. 2nd ed. Oxford University Press.

[ece374146-bib-0065] Sherratt, T. N. 2002. “The Evolution of Imperfect Mimicry.” Behavioral Ecology 13: 821–826.

[ece374146-bib-0066] Sherratt, T. N. 2008. “The Evolution of Müllerian Mimicry.” Naturwissenschaften 95: 681–695. 18542902 10.1007/s00114-008-0403-y PMC2443389

[ece374146-bib-0067] Shirey, V. , R. Khelifa , L. K. M'Gonigle , and L. M. Guzman . 2023. “Occupancy–Detection Models With Museum Specimen Data: Promise and Pitfalls.” Methods in Ecology and Evolution 14: 402–414.

[ece374146-bib-0068] Speed, M. P. 2000. “Warning Signals, Receiver Psychology and Predator Memory.” Animal Behaviour 60: 269–278. 11007635 10.1006/anbe.2000.1430

[ece374146-bib-0069] Speight, M. C. D. , M. C. D. Speight , E. Castella , and J. Sarthou . 2020. “Species Accounts of European Syrphidae, 2020.” Syrph the Net, the Database of European Syrphidae (Diptera).

[ece374146-bib-0070] Stubbs, A. E. , and S. J. Falk . 1983. British Hoverflies. An Illustrated Identification Guide. British Entomological and Natural History Society.

[ece374146-bib-0071] Svensson, B. , J. Lagerlöf , and B. G. Svensson . 2000. “Habitat Preferences of Nest‐Seeking Bumble Bees (Hymenoptera: Apidae) in an Agricultural Landscape.” Agriculture, Ecosystems & Environment 77: 247–255.

[ece374146-bib-0072] Swanson, H. K. , M. Lysy , M. Power , A. D. Stasko , J. D. Johnson , and J. Reist . 2015. “A New Probabilistic Method for Quantifying n‐Dimensional Ecological Niches and Niche Overlap.” Ecology 96: 318–324. 26240852 10.1890/14-0235.1

[ece374146-bib-0073] Swets, J. A. 1988. “Measuring the Accuracy of Diagnostic Information.” Science 80: 1285–1293. 10.1126/science.3287615 3287615

[ece374146-bib-0078] Torvanger, M. S. , Y. L. Dupont , J. M. Olesen , et al. 2025. “The Distribution of Wild Bee Species Along a Latitudinal Gradient in Northern Europe Depends on Their Flower Preferences.” Diversity and Distributions 31, no. 2: e70001.

[ece374146-bib-0074] Van Veen, M. P. 2014. Hoverflies of Northwest Europe: Identification Keys to the Syrphidae. KNNV Uitgeverij.

[ece374146-bib-0075] Warren, D. L. , R. E. Glor , and M. Turelli . 2008. “Environmental Niche Equivalency Versus Conservatism: Quantitative Approaches to Niche Evolution.” Evolution 62: 2868–2883. 18752605 10.1111/j.1558-5646.2008.00482.x

