# Supplementary material for: Do Mimic Hoverflies Spatially Match Their Bumblebee Models? Evidence From a Polymorphic Mimicry System
Source: Ecol Evol. 2026 Aug 11;16(8):e74146. doi: 10.1002/ece3.74146 (PMC13459794; doi:10.1002/ece3.74146)
Supplement: Supplementary file 1 [file ECE3-16-e74146-s001.docx]

**Supplementary Document**

**Supplementary Table S1.** Model evaluation metrics for seven species distribution models using test data. Average and standard deviation (SD) are shown for threshold, AUC, sensitivity, specificity, deviance, Boyce index, and True Skill Statistic (TSS). All models performed well (AUC > 0.85, TSS > 0.7), indicating good discrimination between suitable and unsuitable habitat.

| ***Species*** | **Statistic** | **Threshold** | **AUC** | **Sensitivity** | **Specificity** | **Deviance** | **Boyce** | **TSS** |
| --- | --- | --- | --- | --- | --- | --- | --- | --- |
| ***V. b. plumata*** | Average | 0.349 | 0.978 | 0.983 | 0.899 | 0.762 | 0.452 | 0.882 |
|  | SD | 0.197 | 0.020 | 0.028 | 0.070 | 0.126 | 0.267 | 0.067 |
| ***V. b. haemorrhoidalis*** | Average | 0.570 | 0.867 | 0.894 | 0.884 | 0.577 | 0.427 | 0.778 |
|  | SD | 0.286 | 0.192 | 0.209 | 0.137 | 0.295 | 0.475 | 0.231 |
| ***V. b. bombylans*** | Average | 0.500 | 0.891 | 0.891 | 0.845 | 0.477 | 0.525 | 0.736 |
|  | SD | 0.388 | 0.095 | 0.152 | 0.159 | 0.237 | 0.304 | 0.181 |
| ***B. pascuorum sensu lato*** | Average | 0.508 | 0.999 | 0.995 | 0.996 | 0.973 | 0.626 | 0.991 |
|  | SD | 0.437 | 0.001 | 0.006 | 0.005 | 0.011 | 0.203 | 0.007 |
| ***B. lapidarius sensu lato*** | Average | 0.304 | 0.982 | 0.982 | 0.945 | 0.843 | 0.900 | 0.927 |
|  | SD | 0.166 | 0.028 | 0.024 | 0.068 | 0.110 | 0.047 | 0.067 |
| ***B. pratorum*** | Average | 0.455 | 0.989 | 0.958 | 0.974 | 0.820 | 0.669 | 0.931 |
|  | SD | 0.339 | 0.011 | 0.043 | 0.031 | 0.101 | 0.295 | 0.069 |
| ***B. terrestris sensu lato*** | Average | 0.460 | 0.986 | 0.979 | 0.958 | 0.878 | 0.714 | 0.937 |
|  | SD | 0.288 | 0.017 | 0.025 | 0.057 | 0.080 | 0.296 | 0.056 |

**Supplementary Figure 1:** Compare weighted sampling vs random sampling of the habitat suitability predictions

A. *V. b. plumata*	**B.** *V. b. bombylans*

**C.** *V. b. haemorrhoidalis*	**D.** *B. pascuorum sensu lato*

**E.** *B. lapidarius sensu lato*	**G.** *B. terrestris sensu lato*

**Supplementary Figure 2: Niche size of species pairs :** Posterior distributions of directional ecological niche overlap between bumblebee model species and their respective hoverfly mimics. The plots show the estimated probability that each mimic's niche falls within the niche region of its corresponding model, based on 1,000 posterior samples using the nicheROVER framework. A. *Bombus terrestris sensu latos and Volucella bombylans plumata*; B. *Bombus lapidarius and Volucella bombylans bombylans* and C. *Bombus pascuorum sensu latos and Volucella bombylans* *haemorrhoidalis*. Shaded histograms represent the distribution of overlap probabilities, with vertical lines denoting the mean (solid) and 95% credible intervals (dashed).

**A.**

**B.**

**C.**

NB: _____ **Mean**	----- **95% credible interval**

**Supplementary Fig 3:** Predicted habitat suitability for **A.** *Volucella bombylans plumata* (top) and *Bombus terrestris s.l.* (bottom) **B.** *Volucella bombylans bombylans* (top) and *Bombus lapidarius s.l.* (bottom) **C.** *Volucella bombylans haemorrhoidalis* (top) and Bombus pascuorum s.l. (bottom) across Norway.

NB: Habitat suitability ranges from low (dark blue) to high (yellow).

A.

**B.**

**C.**

**Supplementary Table S2.** Environmental niche overlap metrics between Bumblebee models

| **Model Bumblebee species / Mimic hoverfly species** | **Schoener’s D index** | **Hellinger’s, I index** |
| --- | --- | --- |
| *B. terrestris s. l. / V B. lapidarius s. l.* | 0.93 | 0.95 |
| *B. lapidarius/ B. pascuorum s. l.* | 0.94 | 0.95 |
| *B. terrestris s. l. / B. pascuorum s. l.* | 0.93 | 0.96 |

**Supplementary Table S3.** Environmental niche overlap metrics between also for hoverfly mimics.

| **Mimic hoverfly species** | **Schoener’s D index** | **Hellinger’s, I index** |
| --- | --- | --- |
| *V. b. plumata/ V. b. bombylans* | 0.89 | 0.94 |
| *V. b. bombylans / V. b. haemorrhoidalis* | 0.88 | 0.93 |
| *V. b. plumata / V. b. haemorrhoidalis* | 0.82 | 0.90 |

**Supplementary Figure S4 (Niche Overlap PCA Plots).** Environmental niche overlap among Volucella bombylans colour morphs based on PCA of environmental space: (A) *V. b. plumata* vs *V. b. bombylans,* (B) *V. b. plumata* vs *V. b. haemorrhoidalis*, and (C) *V. b. bombylans* vs *V. b. haemorrhoidalis*.

A. **B.**

**C.**

**Supplementary Table S4. Parameter estimates for competing candidate models (ΔAIC < 2).**

| **Hoverfly morph** | **Candidate model** | **Predictor** | **β ± SE** | **z** | **Wald χ²** | ***P*** |
| --- | --- | --- | --- | --- | --- | --- |
| ***V. b. plumata*** | *B. lapidarius* + *B. pascuorum* | *B. pascuorum* | -0.397 ± 0.719 | -0.552 | 0.30 | 0.581 |
|  |  | *B. lapidarius* | 1.734 ± 0.264 | 6.567 | 43.13 | <0.001 |
|  | *B. lapidarius* + *B. terrestris* | *B. terrestris* | 0.188 ± 0.670 | 0.280 | 0.08 | 0.780 |
|  |  | *B. lapidarius* | 1.636 ± 0.280 | 5.847 | 34.19 | <0.001 |
| ***V. b. bombylans*** | *B. lapidarius* + *B. pascuorum* | *B. pascuorum* | 5.453 ± 9.964 | 0.547 | 0.30 | 0.584 |
|  |  | *B. lapidarius* | 1.584 ± 0.607 | 2.611 | 6.82 | 0.009 |
|  | *B. lapidarius* + *B. terrestris* | *B. terrestris* | 1.074 ± 1.631 | 0.659 | 0.43 | 0.510 |
|  |  | *B. lapidarius* | 1.438 ± 0.688 | 2.089 | 4.36 | 0.037 |
| ***V. b. haemorrhoidalis*** | *B. lapidarius* + *B. pascuorum* | *B. pascuorum* | -0.888 ± 2.081 | -0.427 | 0.18 | 0.670 |
|  |  | *B. lapidarius* | 0.481 ± 0.497 | 0.969 | 0.94 | 0.333 |
|  | *B. lapidarius* + *B. terrestris* | *B. terrestris* | 0.141 ± 1.413 | 0.100 | 0.01 | 0.921 |
|  |  | *B. lapidarius* | 0.397 ± 0.555 | 0.715 | 0.51 | 0.474 |

**Note:** Parameter estimates are presented for competing candidate models within ΔAIC < 2 of the highest-ranked models. Wald χ² values were calculated as $z^{2}$. These models are provided to illustrate the contribution of individual predictors within competing candidate models and complement the model-selection results presented in Table 2.
